# Evolutionarily conserved 12-oxophytodienoate reductase *trans*-lncRNA pair affects disease resistance in tea (*Camellia sinensis*) via the jasmonic acid signaling pathway

**DOI:** 10.1093/hr/uhae129

**Published:** 2024-05-06

**Authors:** Ting Jiang, Tianming Jiao, Yingbang Hu, Tongtong Li, Cheng Liu, Yajun Liu, Xiaolan Jiang, Tao Xia, Li-Ping Gao

**Affiliations:** School of Life Science, Anhui Agricultural University, Hefei 230036 Anhui, China; State Key Laboratory of Tea Plant Biology and Utilization/Key Laboratory of Tea Biology and Tea Processing of Ministry of Agriculture/Anhui Provincial Laboratory of Tea Plant Biology and Utilization, Anhui Agricultural University, Hefei 230036 Anhui, China; School of Life Science, Anhui Agricultural University, Hefei 230036 Anhui, China; State Key Laboratory of Tea Plant Biology and Utilization/Key Laboratory of Tea Biology and Tea Processing of Ministry of Agriculture/Anhui Provincial Laboratory of Tea Plant Biology and Utilization, Anhui Agricultural University, Hefei 230036 Anhui, China; State Key Laboratory of Tea Plant Biology and Utilization/Key Laboratory of Tea Biology and Tea Processing of Ministry of Agriculture/Anhui Provincial Laboratory of Tea Plant Biology and Utilization, Anhui Agricultural University, Hefei 230036 Anhui, China; School of Life Science, Anhui Agricultural University, Hefei 230036 Anhui, China; School of Life Science, Anhui Agricultural University, Hefei 230036 Anhui, China; State Key Laboratory of Tea Plant Biology and Utilization/Key Laboratory of Tea Biology and Tea Processing of Ministry of Agriculture/Anhui Provincial Laboratory of Tea Plant Biology and Utilization, Anhui Agricultural University, Hefei 230036 Anhui, China; School of Life Science, Anhui Agricultural University, Hefei 230036 Anhui, China

## Abstract

Long non-coding RNAs (lncRNAs) have gathered significant attention due to their pivotal role in plant growth, development, and biotic and abiotic stress resistance. Despite this, there is still little understanding regarding the functions of lncRNA in these domains in the tea plant (*Camellia sinensis*), mainly attributable to the insufficiencies in gene manipulation techniques for tea plants. In this study, we designed a novel strategy to identify evolutionarily conserved *trans*-lncRNA (ECT-lncRNA) pairs in plants. We used highly consistent base sequences in the exon-overlapping region between *trans*-lncRNAs and their target gene transcripts. Based on this method, we successfully screened 24 ECT-lncRNA pairs from at least two or more plant species. In tea, as observed in model plants such as *Arabidopsis*, alfalfa, potatoes, and rice, there exists a *trans*-lncRNA capable of forming an ECT-lncRNA pair with transcripts of the 12-oxophytodienoate reductase (*OPR*) family, denoted as the *OPRL/OPR* pair. Considering evolutionary perspectives, the *OPRL* gene cluster in each species likely originates from a replication event of the *OPR* gene cluster. Gene manipulation and gene expression analysis revealed that *CsOPRL* influences disease resistance by regulating *CsOPR* expression in tea plants. Furthermore, the knockout of *StOPRL1* in *Solanum tuberosum* led to aberrant growth characteristics and strong resistance to fungal infection. This study provides insights into a strategy for the screening and functional verification of ECT-lncRNA pairs.

## Introduction

Non-coding RNAs (ncRNAs) play pivotal roles in numerous biological processes across organisms [1–3]. Long non-coding RNAs (lncRNAs) constitute a subclass of ncRNAs characterized by transcripts exceeding 200 nucleotides in length and poor protein-coding potential. They are commonly classified based on their genomic localization and mode of gene expression regulation, either in *cis* or *trans*. For example, lncRNAs can be categorized as natural antisense transcripts (NATs), overlapping lncRNAs (OT-lncRNAs), long intergenic non-coding RNAs (lincRNAs), and intronic non-coding RNAs (incRNAs) [4, 5].

Identifying functional non-coding transcripts from databases containing more lncRNAs poses a significant challenge [6]. LncRNAs exhibit diverse roles in physiological processes within plants, engaging in chromosome silencing, genomic imprinting, chromatin modification, transcriptional activation and interference, control of alternative splicing, regulation of protein translation and transport, and regulation of miRNA function [7]. Several functional roles of lncRNAs in plants have been documented. For example, cold-assisted intronic non-coding RNA (*COLDAIR*) regulates histone methylation within the chromatin region of flowering locus c (*FLC*), a pivotal flowering gene encoding a protein crucial for flowering inhibition, thereby regulating vernalization in *Arabidopsis* [8]. Additionally, the lncRNA *ELENA1*, in conjugation with the mediator of RNA polymerase II transcription subunit 19a-like protein (MED19a), collectively promotes the expression of pathogenesis-related gene1 (*PR1*) in response to pathogenic infections in plants. Mechanistically, *ELENA1* impedes fibrillarin (FIB2) binding to MED19a, facilitating FIB2 release from the *PR1* promoter and augmenting *PR1* expression [9].

Reports on functional lncRNAs in tea plants are rare, with only a few studies using sequencing data for analysis [10, 11]. The unique characteristics of lncRNAs, including weak sequence conservation, low expression levels, strong tissue and organ specificity, and inducible expression, present challenges in accurately estimating their abundance in animals and plants. Moreover, their weak sequence conservation hinders identifying lncRNA functions in non-model plants, such as tea plants, which lack a robust genetic transformation system. Currently, the *Agrobacterium*-mediated transient gene expression system and antisense oligodeoxynucleotide (AsODN)-based gene expression inhibition are widely used for functional verification of lncRNA gene pairs in tea plants [12, 13].

Jasmonic acid (JA) and its derivatives are crucial regulators in plant defense mechanisms against insects and necrotic pathogens [14, 15]. The biosynthesis of JA initiates within the chloroplasts, where the substrate α-linolenic acid (C18:3) undergoes catalysis by 13 lipoxygenases (*13-LOX*), allene oxide synthase (*AOS*), and allene oxide cyclase (*AOC*), leading to the formation of 12-oxo-phytodienoic acid (12-OPDA), an intermediate compound in JA synthesis. Subsequently, 12-OPDA translocates to the peroxisome and is enzymatically converted into JA by the catalytic action of OPDA reductase (*OPR*). *OPR* plays a pivotal role as a critical enzyme in synthesizing JA [16, 17].

Phylogenetic tree analysis facilitates the classification of *OPR* within the various plant populations into seven distinct subgroups [18]. Furthermore, plant *OPRs* can be categorized into two subtypes: OPRI and OPRII. Members of the OPRI subfamily preferentially catalyze *cis*-(−)-OPDA as a substrate, while those of the OPRII subfamily promote the reduction of *cis*-(+)-OPDA [19, 20]. OPRII subfamily members are primarily responsible for JA synthesis in plants. For example, *Arabidopsis OPR3*, belonging to the OPRII subfamily, exhibits a gene mutation resulting in merely 1/500 of the JA content of the wild type (WT), leading to male-sterility phenotypes in *Arabidopsis* [21, 22]. Although OPRI subfamily members are believed to play a secondary role in JA synthesis, they are involved in plant resistance regulation [23]. However, the mechanism by which OPRI subfamily members regulate plant resistance remains unclear. Literature reports suggest that *OPR2*, an OPRI subfamily in *Arabidopsis thaliana*, can convert the direct JA precursor, 4,5-didehydro-JA, into JA, thereby enhancing plant resistance to pathogens and insect feeding [22]. In this study, evolutionary conservative *trans*-lncRNA (ECT-lncRNA) pairs were identified based on conserved sequences in the overlapping regions between lncRNAs and their target genes in tea plants and conserved sequences in the overlapping regions of lncRNAs between tea plants and other plant species. Several *OPRL/OPR* pairs were identified from tea plants, *Arabidopsis*, alfalfa, potato, and rice, in which the *OPR* belonged to the OPRI subfamily. Experimental findings demonstrate that *OPRLs* influence the growth, development, and disease resistance of tea and potato plants subjected to gene manipulation. Overall, this study elucidates the regulatory mechanism of plant OPRI subfamily members and confirms their involvement in regulating plant resistance, growth, and development.

## Results

### Prediction of ECT-lncRNA pairs in plants

To predict the ECT-lncRNA pairs in plants, we first compiled transcript data of lncRNA and mRNAs from stress-treated tea materials in our research group, as detected by a sequencing company. Additionally, transcript data of lncRNAs and mRNAs from various plant species were obtained from public databases, such as CANTATAdb and NCBI. A total of 5101 lncRNAs were identified from 48 000 transcripts from the treated tea seedlings. Through lncRNA–mRNA interaction analysis, 3313 *cis*-lncRNAs and 2904 *trans*-lncRNAs were identified. Detailed information on the identified *cis*-lncRNAs and *trans*-lncRNAs is provided in Supplementary Data Table S1.

Based on the cDNA sequence consistency between lncRNAs and target genes, 24 candidate gene pairs were screened from 2904 tea *trans*-lncRNAs and mRNA data (Table 1). The standard for 24 candidate gene pairs is that there is a highly consistent overlap region of base sequences between lncRNA and target gene exons, and the consistency of base sequences in the overlap region is >80%. These ECT-lncRNA pairs accounted for only 0.8% of the total *trans*-lncRNAs in tea plants. In order to verify the authenticity of ECT-lncRNAs in tea plants, edna we cloned these 24 ECT-lncRNAs, of which 20 were cloned by us (Supplementary Data Fig. S1). Furthermore, following the same prediction procedure, using 24 candidate gene pairs in tea plants as bait sequences for screening, based on the consistency of their overlapping region bases, their orthologous genes were found in at least one plant species (Table 2).

**Table 1:** ECT-lncRNA pairs screened from tea plant genome data.

**LncRNA**		**Target genes**				
		**Target ID**	**Sequence identity with lncRNA (%)**	**Annotation**	**Sequence identity in overlapping region between lncRNAs and their target genes (%)**	**Sequence consistency** ^**a**^
1	*LTCONS_00008922*	*TEA022935.1*	23	Magnesium transporter MRS2–4-like	97	+
2	*LTCONS_00007788*	*TEA012272.1*	29	Eukaryotic initiation factor 4A-2	99	+
3	*LTCONS_00044543*	*TEA004681.1*	22	Zinc finger CCCH domain-containing protein 53 isoform X1	97	+
4	*LTCONS_00026852*	*TEA025907.1*	20	12-Oxophytodienoate reductase	99	+
5	*LTCONS_00004664*	*TEA022107.1*	10	Histone H2B.3	100	−
6	*LTCONS_00016955*	*TEA012518.1*	41	Protein NUCLEAR FUSION DEFECTIVE 6	99	+
7	*LTCONS_00064414*	*TEA033352.1*	25	Elongation factor 1-alpha-like	88	−
8	*LTCONS_00064957*	*TEA000254.1*	23	Transmembrane 9 superfamily member 4-like	91	+
9	*LTCONS_00005358*	*TEA000289.1*	45	Cytochrome b-c1 complex subunit Rieske-4	98	+
10	*LTCONS_00049549*	*TEA007641.1*	14	Vesicle-associated membrane protein 721-like	96	+
11	*LTCONS_00010505*	*TEA009846.1*	17	Dol-P-Man:Man(6)GlcNAc(2)-PP-Dol alpha-1,2-mannosyltransferase	99	−
12	*LTCONS_00051279*	*TEA022308.1*	35	Haloacid dehalogenase-like hydrolase domain-containing protein 3	96	+
13	*LTCONS_00021103*	*TEA008317.1*	14	Alanine—glyoxylate aminotransferase 2 homolog 2 mitochondrial-like	98	+
14	*LTCONS_00041093*	*TEA000506.1*	19	LIMR family protein At5g01460	92	+
15	*LTCONS_00041460*	*TEA024759.1*	20	Proteasome subunit alpha type-1-B	100	−
16	*LTCONS_00049579*	*TEA022706.1*	37	GDSL esterase/lipase	100	+
17	*LTCONS_00063928*	*TEA008207.1*	16	Peptidyl-prolyl cis-trans isomerase NIMA-interacting 4-like isoform X1	85	+
18	*LTCONS_00002846*	*TEA012270.1*	43	Calmodulin-like	97	+
19	*LTCONS_00007220*	*TEA005414.1*	31	40S ribosomal protein S6	91	+
20	*LTCONS_00037250*	*TEA004050.1*	39	Mitochondrial outer membrane protein porin of 36 kDa	100	+
21	*LTCONS_00050008*	*TEA010642.1*	12	Basic leucine zipper 34-like	99	+
22	*LTCONS_00050164*	*TEA015820.1*	21	BI1-like protein	89	−
23	*LTCONS_00053896*	*TEA032488.1*	29	2-Alkenal reductase (NADP(+)-dependent)-like	98	+
24	*LTCONS_00053641*	*TEA028539.1*	35	14-3-3-like protein	99	+

^a^+, Consistency; −, complementarity.

**Table 2:** ECT-lncRNA pairs screened from other species.

	**LncRNA from tea plants**	**Orthologous lncRNA (species)**	**Target genes of species**			
			**Target ID**	**Sequence identity with lncRNA (%)**	**Sequence identity of overlapping region (%)**	**Sequence consistency** ^**a**^
*1*	*LTCONS_00008922*	*CNT2038389* (*Malus domestica*)	*XM_029094701.1*	31	99	−
		*CNT2092531* (*Solanum tuberosum*)	*XM_006349290.2*	20	100	+
*2*	*LTCONS_00007788*	*CNT20227177* (*Theobroma cacao*)	*XM_007012261.2*	39	100	−
		*CNT2072931* (*Populus trichocarpa*)	*XM_024604078.1*	18	100	−
		*CNT2080641* (*Oryza nivara*)	*XM_026026114.1*	45	100	−
*3*	*LTCONS_00044543*	*CNT20189851* (*Prunus persica*)	*XM_020556848.1*	13	98	−
*4*	*LTCONS_00026852*	*CNT20208736* (*Medicago truncatula*)	*XM_003610710.4*	31	98	−
		*CNT20208735* (*Medicago truncatula*)		31	98	−
		*CNT2091126* (*Solanum tuberosum*)	*XM_006367626*	57	94	−
		*CNT20180321* (*Arabidopsis thaliana*)	*NM_106318*	19	97	−
		*CNT20229132* (*Oryza sativa*)	*AK108079*	20	97	−
*5*	*LTCONS_00004664*	*CNT2033313* (*Glycine max*)	*NM_001360367.2*	59	100	−
		*CNT2047077* (*Chenopodium quinoa*)	*XM_021860512.1*	38	96	−
		*CNT20237064* (*Vitis vinifera*)	*XM_019220518.1*	46	95	−
*6*	*LTCONS_00016955*	*CNT2043167* (*Malus domestica*)	*XM_008383619.2*	37	100	+
		*CNT2043166* (*Malus domestica*)	*XM_008383620.3*	45	100	+
		*CNT2043165* (*Malus domestica*)	*XM_008383621.3*	19	97	+
		*CNT2043164* (*Malus domestica*)		36	100	+
		*CNT2043163* (*Malus domestica*)		42	100	+
		*CNT2039564* (*Malus domestica*)	*XM_008383620.3*	36	100	+
		*CNT2038026* (*Malus domestica*)	*XM_008379084.3*	42	100	+
		*CNT2038025* (*Malus domestica*)	*XM_008379085.3*	45	100	+
*7*	*LTCONS_00064414*	*CNT2035352* (*Malus domestica*)	*XM_008382323.3*	44	96	−
		*CNT20171807* (*Brassica oleracea*)	*XM_013748622.1*	53	100	−
		*CNT2044767* (*Malus domestica*)	*XM_029099752.1*	32	100	−
		*CNT20145755* (*Brassica napus*)	*XM_013860892.3*	44	97	−
		*CNT208893* (*Cucumis sativus*)	*XM_004138916.3*	69	97	−
		*CNT208892* (*Cucumis sativus*)		61	97	−
		*CNT207653* (*Cucumis sativus*)		59	94	−
		*CNT205932* (*Cucumis sativus*)	*XM_051090445.1*	48	98	−
*8*	*LTCONS_00064957*	*CNT2074390* (*Populus trichocarpa*)	*XM_006384061.3*	50	100	−
		*CNT2074389* (*Populus trichocarpa*)		44	100	−
		*CNT2038576* (*Malus domestica*)	*XM_008376813.3*	25	100	−
*9*	*LTCONS_00005358*	*CNT20111926* (*Brassica rapa*)	*XM_009127664.3*	62	97	−
		*CNT20143858* (*Brassica napus*)	*XM_003757908.1*	58	98	−
*10*	*LTCONS_00049549*	*CNT20116043* (*Brassica rapa*)	*XM_009134663.3*	31	100	−
		*CNT20149407* (*Brassica napus*)	*XM_013878762.3*	21	91	−
*11*	*LTCONS_00010505*	*CNT2042500* (*Malus domestica*)	*XM_029089082.1*	35	95	+
		*CNT2038101* (*Malus domestica*)		21	98	−
*12*	*LTCONS_00051279*	*CNT2044449* (*Malus domestica*)	*XM_008394181.3*	23	97	+
		*CNT2044448* (*Malus domestica*)		28	97	+
*13*	*LTCONS_00021103*	*CNT20237770* (*Vitis vinifera*)	*XM_002267751.4*	27	98	−

(Continued)

**Table 2:** Continued

	**LncRNA from tea plants**	**Orthologous lncRNA (species)**	**Target genes of species**			
			**Target ID**	**Sequence identity with lncRNA (%)**	**Sequence identity of overlapping region (%)**	**Sequence consistency** ^**a**^
*14*	*LTCONS_00041093*	*CNT2037034* (*Malus domestica*)	*XM_008357492.3*	21	100	+
*15*	*LTCONS_00041460*	*CNT20238040* (*Vitis vinifera*)	*XM_002281912.3*	31	100	−
*16*	*LTCONS_00049579*	*CNT2043334* (*Malus domestica*)				
*17*	*LTCONS_00063928*	*CNT2028772* (*Manihot esculenta*)	*XM_021786738.1*	13	96	+
*18*	*LTCONS_00002846*	*CNT20180106* (*Arabidopsis lyrata*)	*XM_021024651.1*	43	100	−
*19*	*LTCONS_00007220*	*CNT2075529* (*Populus trichocarpa*)	*XM_006382832.3*	29	100	−
*20*	*LTCONS_00037250*	*CNT2072920* (*Populus trichocarpa*)	*XM_006378092.3*	61	100	−
*21*	*LTCONS_00050008*	*CNT2030544* (*Manihot esculenta*)	*XM_021763170.2*	30	100	+
*22*	*LTCONS_00050164*	*CNT20237038* (*Vitis vinifera*)	*XM_002279332.4*	31	100	−
*23*	*LTCONS_00053896*	*CNT2072341* (*Populus trichocarpa*)	*XM_035033563.1*	17	96	−
*24*	*LTCONS_00053641*	*CNT20238707* (*Vitis vinifera*)	*XM_002262766.4*	26	100	−

* +, Consistency; −, complementarity.

NATs are coding or non-coding RNAs with sequence complementarity to other transcripts (sense transcripts), potentially regulating the expression of their sense partner(s) at either the transcriptional or post-transcriptional level. Depending on their genomic origins, NATs can be classified into *cis*-acting and *trans*-acting NATs [24, 25]. While *cis*-NATs have been extensively studied, research on *trans*-NATs is still rare [26–28]. In the 24 ECT-lncRNA pairs and 49 homologous ECT-lncRNA pairs, there are 5 and 37 *trans-*NAT pairs in tea plants (Table 1) and other plants (Table 2), respectively. Even among the homologous ECT-lncRNA pairs, some may be *trans-*NAT pairs, while others are not, e.g. 12-oxophytodienoate reductase *trans*-lncRNA pairs.

Sequence alignment results revealed very low cDNA sequence consistency between *trans*-lncRNAs and their target genes (10–45%) in tea plants. However, the sequence consistency of the overlapping region between *trans*-lncRNAs and their target genes was very high (85–100%) (Table 1). Similarly, the sequence alignment results showed very low sequence consistency between orthologous *trans*-lncRNAs and their target genes, ranging from 13% to 69%. Nevertheless, in the overlapping region the sequence consistency between *trans*-lncRNAs and their target genes was very high, ranging from 91 to 100% (Table 2).

Functional enrichment analysis of the 24 ECT-lncRNA pairs revealed that genes involved in plant growth and development included magnesium transporter MRS2-4-like, eukaryotic initiation factor 4A-2, zinc finger CCCH domain-containing protein, and calmodulin-like proteins. Additionally, genes associated with plant resistance response included 12-oxophytodienoate reductase, cytochrome b-c1 complex subunit Rieske-4, vesicle-associated membrane protein, calmodulin-like proteins, and 14-3-3-like proteins (Table 1).

### Evolutionary conservation of *OPRL/OPR* pairs in plants

In the preceding results, our focus lies on the evolutionarily conserved 12-oxophytodienoate reductase–*trans*-lncRNA (*OPRL*-trans-lncRNA) pairs present in tea plants, *Arabidopsis*, potato, alfalfa, and monocotyledonous rice, where the mRNA transcript in these pairs is predicted to encode *OPRs*, pivotal components of the jasmonate signaling pathway. These 12-oxophytodienoate reductase–*trans*-lncRNA pairs are hereafter referred to as *OPRLs/OPRs*.

The gene sequences and chromosomal locations of *OPRs* and *OPRLs*, spanning algae, gymnosperms, and angiosperms, were acquired from genomic data provided by NCBI (Supplementary Data Tables S2 and S3). While *OPRs* were detected across algal, gymnosperm, and angiosperm genomes, *OPRLs* were exclusively identified in angiosperms, including monocotyledonous rice, dicotyledonous *Arabidopsis*, alfalfa, horseradish, potato, and tea tree. No responsive *OPRLs* were observed in algae and gymnosperms.

**Figure 1:**
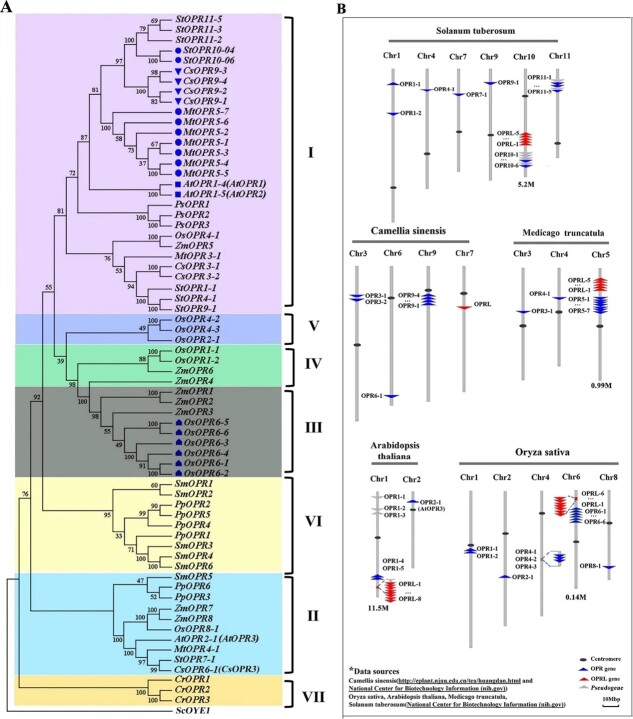
Phylogenetic analysis of *OPR* genes in 10 representative plants and chromosome mapping of five pairs of 12-oxophytodienoate reductase (*OPR*) and *OPRL.*  **A** Neighbor joining and minimalist analysis methods were used to construct a phylogenetic tree using the MEGA5.0 software. The tree consists of seven groups, each highlighted in a different color. Sequence information is provided in Supplementary Data Table S2. **B** Chromosome localization of *OPR*/*OPRL* pairs in monocotyledons (*Oryza sativa*) and dicotyledons (*Solanum tuberosum*, *Camellia sinensis*, *Medicago truncatula*, and *Arabidopsis thaliana*). Location information is provided in Supplementary Data Table S3.

According to the phylogenetic analysis, the *OPR* family was divided into seven subgroups across algae, gymnosperms, and angiosperms (Fig. 1A). Within tea plants, the seven identified *OPR* genes were distributed between the first (belonging to the OPRI subfamily) and second subgroups (belonging to the OPRII Subfamily). Chromosome mapping revealed that *OPR*s and *OPRL*s in tea plants, rice, *Arabidopsis*, alfalfa, and potato were dispersed across chromosomes (Fig. 1B). Notably, four *OPR*s in tea plants (*CsOPR9-1*, *CsOPR9-2*, *CsOPR9-3*, and *CsOPR9-4*) were found in tandem on chromosome 9, with *CsOPR6-1*, a member of the OPRII subfamily member (*CsOPR6-1*), a direct homolog of *AtOPR3*, located on chromosome 6. *CsOPR9-1/2/3/4*, members of the OPRI subfamily, constitute the OPR portion of the *OPRL/OPR* pairs. Similarly, OPR genes in other plants are either clustered or individually distributed on different chromosomes. In contrast, the *OPR* component of the *OPRL/OPR* pairs is situated on chromosomes as gene clusters. Thus, the *OPR* portion of the *OPRL/OPR* pairs in various plants belongs to the OPRI subfamily and is organized as gene clusters on chromosomes.

**Figure 2:**
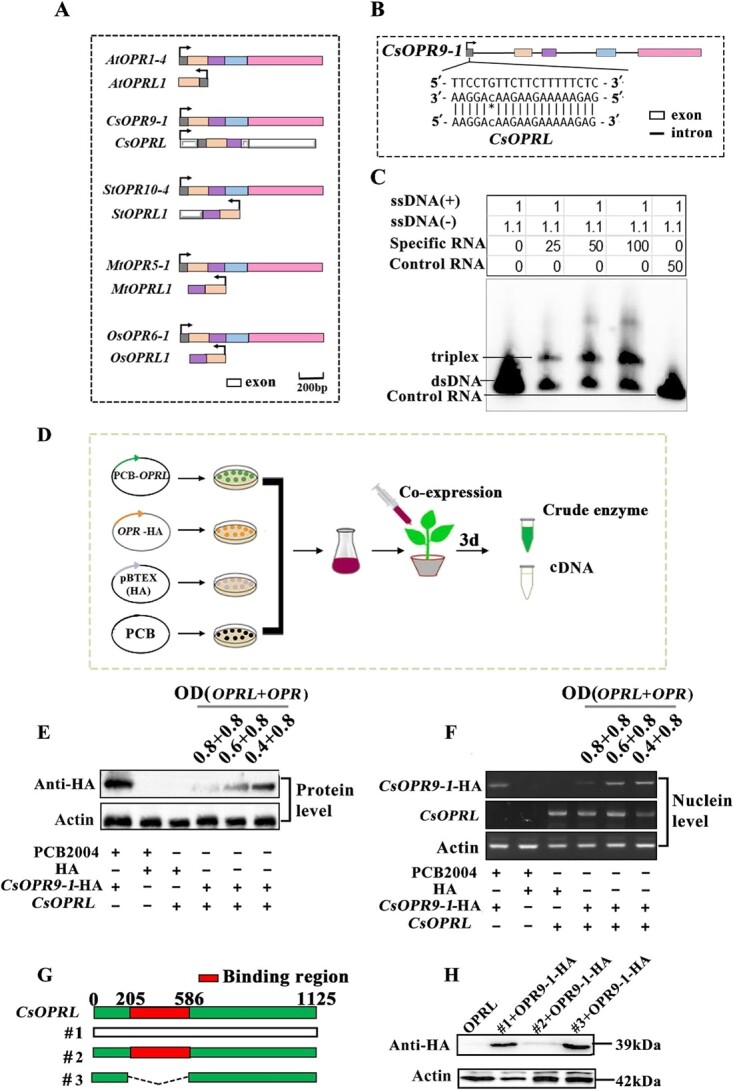
Sequence analysis of *OPRL/OPR* pairs and identification of mechanisms through which *CsOPRL* regulates *CsOPR9-1.*  **A** Structure diagram of *OPRL/OPR* pairs in five plants. **B**, **C** Site of formation of RNA–DNA triplexes in tea plants and gel formation map of EMSA *in vitro*. **D** Co-expression of *OPRL/OPR* pairs. **E**, **F** Western blotting and RT–PCR analysis of the interaction between *OPR*s and *OPRL*s in tea plants. **G**–**H**  *CsOPRL* was truncated and recombined to further validate its key functional region.

Chromosome mapping unveiled that *OPRLs* were arranged on chromosomes as tandem gene clusters comprising five to eight *OPRL* genes in all plants except the tea plant. The *OPRL* clusters in all plants, excluding tea plants, and their target *OPR* clusters were positioned adjacent to the same chromosome. The distance between *OPR*s and *OPRL*s varied from 0.14 to 11.5 Mb across different plants. Moreover, the length of *OPRL* sequences exhibited inconsistency among plants, with the shortest sequence identified in *A. thaliana* (227 bp) and the most extended sequence observed in the tea plant (*CsOPRL*; 1125 bp). Utilizing sequence alignment results (Supplementary Data Fig. S2), each *OPRL* was paired with its corresponding target *OPR* to form an *OPRL/OPR* pair (Supplementary Data Table S4). Notably, based on highly conserved overlapping region sequences, *CsOPRL* was found to form *OPRL/OPR* pairs with *CsOPR9-1/2/3/4*. Furthermore, the alignment results indicated that the base sequences in the overlapping regions of eight *AtOPRLs* were entirely consistent with those of *AtOPR1-4* (*AtOPR1*) and exhibited high consistency (96%) with *AtOPR1-5* (*AtOPR2*) and relatively low sequence consistency (40%) with *AtOPR2-1* (*AtOPR3*). This suggests that *AtOPRL*s might regulate the function of *AtOPR1* and *AtOPR2* rather than *AtOPR3*. Sequence alignment was conducted between *OPR*s and *OPRL*s to determine the location of the overlapping region in *OPRL/OPR* pairs (Supplementary Data Fig. S3) (Fig. 2A). In *A. thaliana*, eight *AtOPRL*s overlapped with *AtOPR1* or *AtOPR2* at the N-terminus, with the overlapping region situated in the first and second exon regions spanning the *AtOPR1* transcript or *AtOPR2* transcript. It is worth noting that there are no introns in the DNA sequence of the *AtOPRL* genes (Supplementary Data Table S3), unlike the DNA sequence of the *AtOPR1* or *AtOPR2* genes.

In *Medicago truncatula*, five *MtOPRL*s and seven *MtOPR*s were found to overlap at the N-terminus. The overlapping region encompassed the second and third exons of two *MtOPR*s and exhibited high conservation (95%). Similarly, in *Solanum tuberosum*, five *StOPRL*s and two *StOPR*s overlapped in the second and third exon regions at the N-terminus, with a sequence similarity of 90%. Additionally, the second and third exon regions of six *OsOPRL*s and six *OsOPR*s overlapped at the N-terminus in *Oryza sativa*, with a sequence similarity of 92%. In the tea plant, *OPRL* overlapped with *OPR9-1/2/3/4* at the N-terminus, spanning the first, second, and third exon regions. A schematic diagram illustrating the overlapping regions between *OPR*s and *OPRL*s in different plants is presented in Fig. 2A. Given the similarity of these overlapping regions and their comparable chromosomal locations across different plants, it is plausible that the *OPRL*/*OPR* pairs are conserved in angiosperms.

To confirm the reliability of the *OPRL* sequences mentioned above, we cloned the DNA sequence of *OPRL* from the tea plant. We discovered that the overlapping region between *OPRL* and *OPR9-1/2/3/4* contained the first, second, and third exons and an intron sequence (Supplementary Data Fig. S3).

Based on these findings, we hypothesize that the *OPRL* clusters in plants may originate from incomplete residues of *OPR* clusters following replication, potentially representing pseudogenes with impaired functions or newly functionalized genes.

### Mechanism underlying the interaction between *CsOPRL* and *CsOPR9-1*

We hypothesize that the single-stranded RNA of *OPRL*s and one of the two DNA strands of *OPR*s form a complementary chain in the overlapping region, thus creating a triplex that impedes the transcription of the target *OPR*s. To test this hypothesis, Triplexator [29], a predictor of RNA–RNA and RNA–DNA interactions, was utilized to predict the sites where *OPR*s and *OPRL*s may form triple helices in different species. However, the results revealed inconsistency in the sites where *OPR*s and *OPRL*s formed triple helices among species (Supplementary Data Fig. S1).

A 20-bp sequence in the overlying region of Cs*OPRL* predicted to form a triplex with CsOPR9-1 was synthesized to validate the prediction accuracy (Fig. 2B). The presence of this triplex was confirmed in samples containing specific RNA and the sense and antisense strands of DNA of *CsOPR9-1*, with its content increasing proportionally with RNA concentration (Fig. 2C). These findings suggest that Cs*OPRL* can bind to the DNA of *CsOPR9-1*, thereby suppressing its transcription.

To confirm the regulatory effects of *CsOPRL* on *CsOPR9-1* expression *in vivo*, we constructed two expression vectors encoding *CsOPR9-1*-HA and *CsOPRL*, respectively, and transiently co-expressed them in *N. benthamiana* using the cauliflower mosaic virus (CaMV)-*35S* promoter (Fig. 2D). Western blotting and semi-quantitative RT–PCR revealed a gradual increase in the protein and mRNA expression of *CsOPR9-1*-HA with decreasing *CsOPRL* concentration (Fig. 2E and F). These results suggest that *CsOPRL* inhibits the expression of *CsOPR9-1* and affects its protein expression.

To identify the site of action of *CsOPRL*, the overlapping region (205th–586th base) of *CsOPRL* was truncated (Fig. 2G). Subsequently, empty (1#), complete *CsOPRL* (2#), and truncated *CsOPRL* (3#) expression vectors were co-expressed with *CsOPR9-1*-HA in *N. benthamiana* using the *35S* promoter. Western blotting revealed the absence of *CsOPR9-1*-HA in the processing area with the overlapping region (*CsOPRL* [2#]) (Fig. 2H). These results indicate that the inhibitory effects of *CsOPRL* on *CsOPR9-1*-HA expression were attenuated after the overlapping region was truncated, suggesting that *CsOPRL* and *CsOPR9-1* interact in the overlapping region.

### Confirmation of the regulatory effects of *CsOPRL* on *CsOPR9-1* function using a heterologous expression system

To verify the function of *CsOPR9-1*, the *35S* promoter of the *CaMV* was used to induce heterologous overexpression of *CsOPR9-1* and *CsOPRL* in the model plant *A. thaliana*. Subsequently, plants co-expressing *CsOPR9-1* and *CsOPRL* were obtained through hybridization (Supplementary Data Fig. S4). RT–PCR analysis revealed a significant reduction in the transcriptional level *CsOPR9-1* in plants co-expressing *CsOPR9-1* and *CsOPRL*.

**Figure 3:**
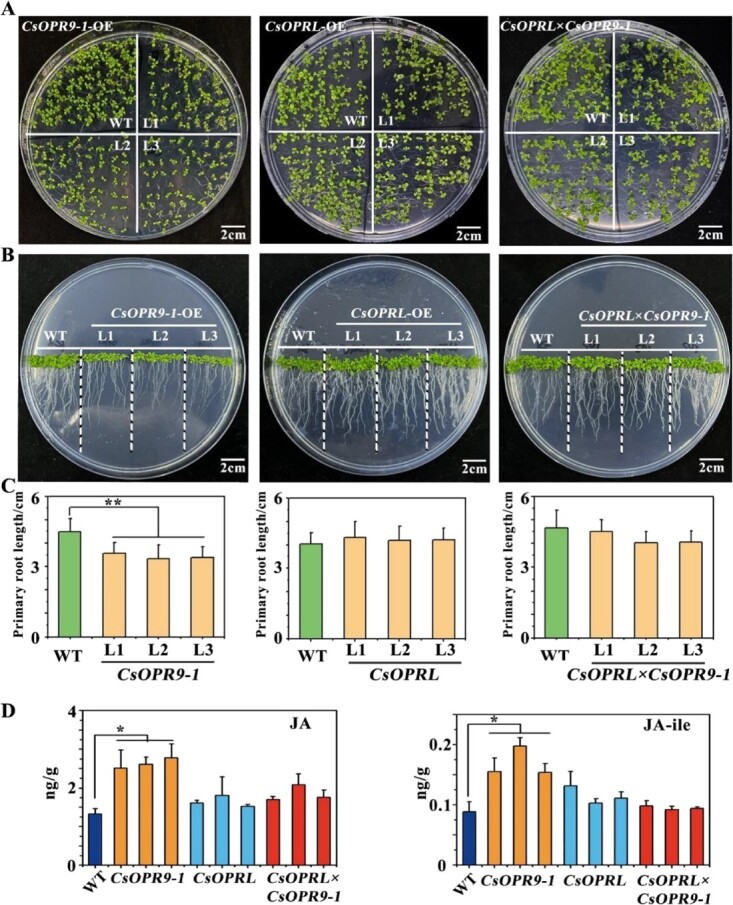
Exogenous overexpression of *CsOPRL* can restore the inhibitory effects of *CsOPR9-1* on plant development in *A. thaliana*. **A**, **B** Growth phenotypes of the primary leaves and roots of 7-day-old *Arabidopsis* seedlings (scale bar = 2 cm). **C**, **D** Physiological indicators and JA and JA-ile contents of *CsOPRL*, *CsOPR9-1*, and *CsOPRL × CsOPR9-1*.

Morphological observations of plant cultures indicated that *CsOPR9-1*-overexpressing plants exhibited significantly smaller sizes and shorter root lengths than the WT, indicating inhibited growth (Fig. 3A–C). However, these characteristics did not significantly differ among control plants, plants expressing *CsOPRL* alone, and plants co-expressing *CsOPRL* and *CsOPR9-1*. Subsequently, we assessed the JA and JA isoleucine (ile) contents in *Arabidopsis*. We observed a significant increase in JA and JA-ile contents in *CsOPR9-1*-overexpressing plants compared with control plants. In contrast, no significant change was observed in JA and JA-ile in *CsOPRL*-overexpressing and hybrid plants (Fig. 3D).

### Confirmation of *CsOPR9-1*-induced increase in resistance to pathogenic fungal infection in tea plants

The JA pathway is known to play a pivotal role in the resistance of tea plants to fungal infection [30, 31]. *OPR*s serve as crucial enzymes catalyzing the synthesis of JA, which branches into two pathways: the *OPR3*-0dependent main pathway and the *OPR2*-catalyzed *OPR3*-independent alternative pathway [22].

**Figure 4:**
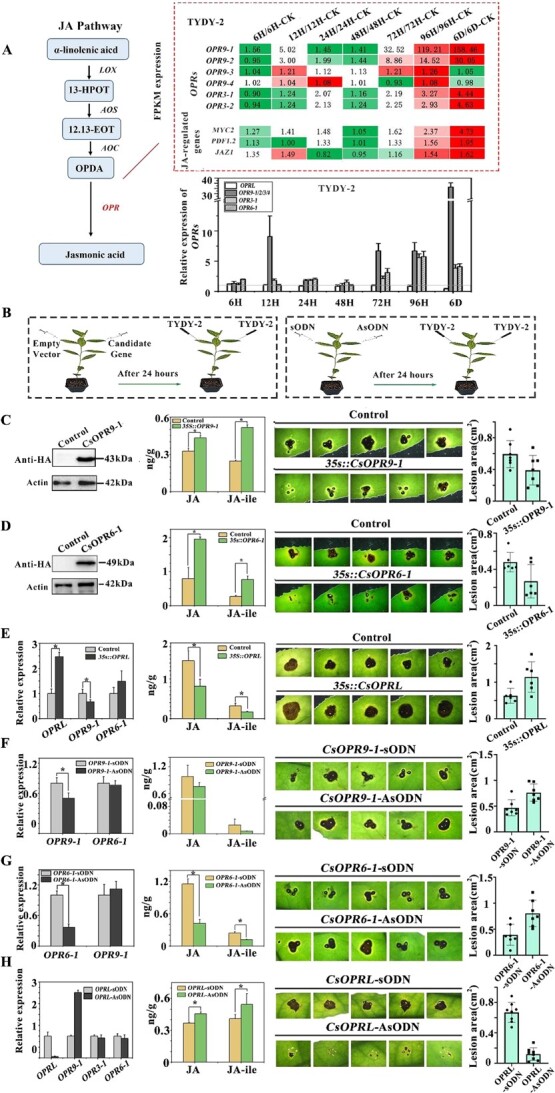
Role of *CsOPRs* and *CsOPRL* in resistance of tea plants to anthracnosis. **A** Schematic diagram of *CsOPR*s in the jasmonic acid pathway. Changes in *CsOPR* and JA marker gene expression were examined in leaves with anthracnosis via transcriptomic sequencing and fluorescence quantitative detection. **B** Effects of transient gene expression mediated by *Agrobacterium tumefaciens* and gene silencing mediated by AsODNs on the resistance of tea plants to anthracnosis. **C**–**E** Effects of *Agrobacterium*-mediated transient expression of *CsOPR*s and *CsOPRL* on the resistance of tea plants to anthracnosis and accumulation patterns of JA and JA-ile in tea plants. The recording time of lesion size was 6 dpi (scale bar = 2 mm). **F**–**H** Effects of gene silencing induced by AsODNs on the resistance of tea plants to anthracnosis and accumulation patterns of JA and JA Ile in tea plants. The recording time of lesion size was 6 dpi (scale bar = 2 mm).

Anthracnosis was induced in tea leaves by injecting them with the TYDY-2 strain, and transcriptome sequencing was used to assess the expression of *CsOPR*s. Four *OPR*s detected were significantly upregulated: *CsOPR9-1*, *CsOPR9-2*, *CsOPR3-1*, and *CsOPR3-2*. On day 6 of inoculation, the expression of these genes was 158.45, 30.04, 4.44, and 4.63 times higher compared with the control group. Concurrently, several JA marker genes, such as *MYC2*, *PDF1.2*, and *JAZ5*, were also upregulated to varying extents (Fig. 4A). These findings suggest an upregulation of the JA synthesis pathway in tea plants with anthracnosis, predominantly evidenced by the upregulation of *CsOPR9-1*, the target gene of *CsOPRL*. Additionally, qPCR analysis revealed upregulation of *CsOPR9s*, *CsOPR3-1*, and *CsOPR6-1*, with *CsOPR9-1* showing notably higher expression than the others. In contrast, *CsOPRL* expression was downregulated (Fig. 4A). It is worth noting that *CsOPR6-1* upregulated gene expression 5-fold after 6 days of inoculation, while *CsOPR9-1* was upregulated by as much as 35-fold.

The effects of exogenous methyl jasmonate (MeJA) and the JA synthesis inhibitor diethyldithiocarbamic acid (DIECA) on tea plants were examined to validate the role of JA in enhancing tea plant resistance to anthracnose (Supplementary Data Fig. S5A). The results demonstrated that exogenous MeJA significantly reduced the size of infected spots on leaves. In contrast, the JA synthesis inhibitor DIECA increased infected spots, indicating the crucial role of the JA pathway in tea plant resistance to fungal infection. Supplementary Data Fig. S5B displays the effect of MeJA treatment and JA synthesis inhibitor DIECA on the expression of *CsOPR* and *CsOPRL* genes in tea plants using fluorescence. The results showed that exogenous MeJA treatment promoted the expression of *CsOPR9*s, *CsOPR3-1*, and *CsOPR6-1*, with the upregulation of *CsOPR9-1* expression earlier than the promotion of *CsOPR3-1* and *CsOPR6-1*. Conversely, exogenous MeJA treatment inhibited the expression of *CsOPRL*, while the effects of JA synthesis inhibitor DIECA were the opposite. This result indicates that the expression of the *CsOPRL* gene is regulated by JA levels in tea plants.

To elucidate the roles of *CsOPR9-1*, *CsOPR6-1*, and *CsOPRL* genes in tea plant resistance to anthracnose, we used *Agrobacterium*-mediated transient overexpression technology and antisense oligonucleotide-mediated gene silencing technology (Fig. 4B). *Agrobacterium* strains containing *CsOPR9-1*-HA, *CsOPR6-1*-HA, and *CsOPRL* genes were injected into tea leaves, followed by inoculation with the TYDY-2 strain to induce disease. Subsequently, we assessed the expression levels of corresponding genes and proteins by immunoblotting with HA antigen (Fig. 4C–E). Our results revealed a significant increase in the expression level of *CsOPR9-1* in the experimental group compared with the control, accompanied by a slight increase in JA and JA-ile content and a significant reduction in the area of leaf spots (Fig. 4C). Moreover, the accumulation of JA and JA-ile in plants transiently expressing *CsOPR6-1* was significantly higher than that in plants transiently overexpressing *CsOPR9-1* (Fig. 4D), potentially due to *CsOPR6-1*’s involvement in the main pathway of JA synthesis in tea plants. Additionally, in tea plant leaves transiently overexpressing *CsOPRL*, the expression level of the *CsOPR9-1* gene was significantly downregulated. In contrast, *CsOPR6-1* expression remained largely unaffected (Fig. 4E). This suggests that *CsOPRL* modulates *CsOPR9-1* expression without affecting *CsOPR6-1*. Furthermore, in tea plant leaves transiently overexpressing *CsOPRL*, compared with the control group, the content of JA and JA-ile decreased, and the area of leaf spots on the leaves increased significantly (Fig. 4E).

The gene expression inhibition test results mediated by AsODNs yielded anticipated outcomes. Specifically, when the expression of *CsOPR9-1* and *CsOPR6-1* genes was interfered with, the experimental group exhibited significantly larger lesion spots compared with the control group, accompanied by a decrease in the contents of JA and JA-ile (Fig. 4F and G). Conversely, interfering with the expression of *CsOPRL* led to a significant increase in the expression level of *CsOPR9-1*. In contrast, the expression levels of *CsOPR6-1* and *CsOPR3-1* genes remained relatively unchanged, further indicating that *CsOPR9-1* is the target of *CsOPRL* in tea plants, whereas *CsOPR6-1* and *CsOPR3-1* are not (Fig. 4H). In tea seedlings in which the expression of *CsOPRL* was interfered with, the contents of JA and JA-ile slightly increased, but the area of leaf spots on the leaves decreased significantly compared with the control group (Fig. 4H). Additionally, genes involved in the JA synthesis pathway, including *LOX*, *AOS*, and *AOC*, exhibited upregulation (Supplementary Data Fig. S6).

These findings suggest that the JA pathway plays a role in enhancing the resistance of tea plants to fungal infection. Moreover, *CsOPRL* contributes to anthracnose resistance by modulating the expression of *CsOPR9-1* and influencing the synthesis of JA.

### Confirmation of *StOPRL* function in *S. tuberosum*

The function of *StOPRL* was determined in *S. tuberosum* to determine whether *OPRL/OPR* pairs exhibit similar functions across different plant species.

**Figure 5:**
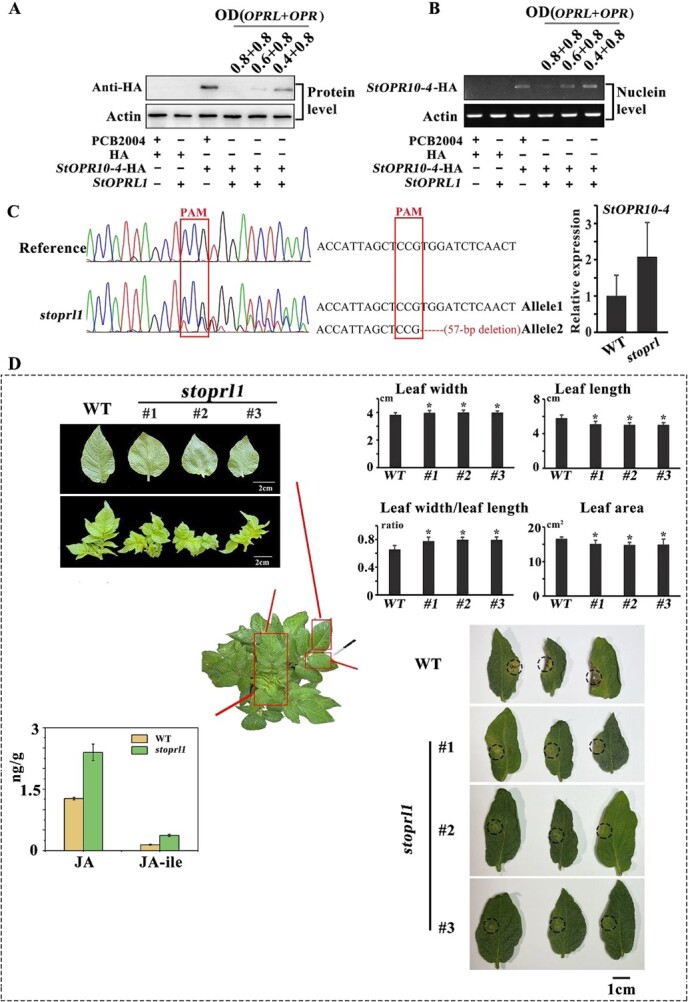
Role of *StOPRL1* in the growth and development of potato plants and their resistance response to anthracnosis. **A**, **B** Western blotting and RT–PCR analysis of the interaction between *OPR* and *OPRL* in potato plants. **C** Cas9-mediated knockout and *StOPR10-4* expression analysis of *StOPRL1* in potatoes. **D** Physiological indicators, JA and JA-ile contents, and disease resistance in the potato knockout line *stoprl1*.

*StOPRL1* and its target gene, *StOPR10-4,* were cloned for subsequent functional validation. The findings revealed that *StOPRL1* suppressed the expression of *StOPR10-4* and decreased its protein content (Fig. 5A and B).

Furthermore, *StOPRL*-knockout plants (*stoprl1*) were produced using Cas9 technology to verify the function of *StOPRL*1. Quantitative real-time PCR (qRT–PCR) results demonstrated a 1-fold increase in the expression of *StOPR10-4* in *stoprl1* plants (Fig. 5C). The primary leaves of *stoprl1* plants displayed enhanced curling and shrinking, accompanied by near accumulation of JA and JA-ile. Additionally, the terminal lobule exhibited shorter leaf length, increased width, and smaller leaf area. Compared with plants with WT, *stoprl1* plants exhibited significantly higher resistance to anthracnosis and a smaller lesion area (Fig. 5D), However, since the *stoprl1* mutant strains in this repeat experiment showed almost no disease, only a slight yellowing phenotype, it was not possible to measure the lesion area. These findings suggest that *StOPRL*, similar to *CsOPRL* in tea plants, exerts a regulatory role in plant resistance.

## Discussion

### Identification of *OPRL/OPR* pairs in angiosperms

From an evolutionary perspective, *OPR* genes exhibit a relatively ancient origin, being distributed across various plant groups, including green algae, bryophytes, lycopene-rich plants, gymnosperms, monocotyledonous plants, and dicotyledonous plants. However, the expansion of the *OPR* family in terrestrial plants has occurred extensively through diverse mechanisms, such as cascading repetitive events [18]. Our study revealed through phylogenetic tree analysis that *OPR* genes experiencing tandem repeat gene amplification events are primarily concentrated within the first and third subgroups, forming gene clusters on chromosomes (Fig. 1A).

Sequence alignment between *OPR*s and *OPRL*s and their chromosomal locations suggests that the identified group of *OPRL* clusters may have originated from residual *OPR* genes following replication events (Fig. 1B). The conservation of *OPRL/OPR* pairs across angiosperms, observed in dicotyledonous plants like *Arabidopsis*, tea, alfalfa, and potatoes, and monocotyledonous plants like rice, is noteworthy. *OPRL*s exhibit a clustered distribution in the genomes of most studied plants, except for tea plants, which are located on the same chromosome as the *OPR* gene cluster (Fig. 1B). This suggests a replication event of the *OPR* gene cluster may be on the same chromosome, possibly resulting in partial sequence loss of the gene. Mosses, lycophytes, and gymnosperms do not display similar *OPRL* clusters. Overall, these findings imply that replication events leading to the formation of the *OPR* gene cluster likely occurred before the differentiation of monocotyledons and dicotyledons.

Functional modifications after gene replication, such as pseudogene formation [32, 33], sub-functionalization [34], new functionalization [35], and sub-neural functionalization [36], may have altered the functional constraints between gene clusters within the gene family. The observed sequence consistency between *OPRL*s and *OPR*s may have originated from *OPR* replication events and subsequently undergone new functionalization.

### *OPRL* demonstrates the regulatory role of *OPR* target gene

From an evolutionary perspective, *OPR* family genes undergo varying degrees of variation in their introns, exons, and critical amino acid residues in response to long-term selective stress. These changes inevitably impact the function of *OPR* genes, resulting in subgroup-specific functions [18].

*OPRs* can be classified into OPR-I and OPR-II based on their substrate specificity in *Arabidopsis* [22]. Studies have shown that *AtOPR*s belonging to subfamily I preferentially catalyze 9*R*,13*R*-OPDA [37], whereas *AtOPR2-1* belonging to subfamily II catalyzes the reduction of 9*S*,13*S*-OPDA to form OPC 8:0 [22, 38]. Additionally, *OPR* members of subfamily II may participate in the JA biosynthesis pathway, while those in subfamily I may be involved in the defense signaling pathway. Subfamily III is exclusive to monocotyledonous plants and likely plays a crucial role in defense signaling and mitogen-activated protein kinase pathways [39].

The *OPRs* investigated in this study belong to subfamilies I and III. Gene manipulation experiments in tea plants demonstrated that *CsOPR9-1* and *CsOPRL* are involved in regulating the resistance of tea plants to anthracnosis (Fig. 4). Additionally, *OPRL* knockout resulted in abnormal growth and altered resistance response in potato plants (Fig. 5), suggesting that *OPRL* regulates plant growth, development, and resistance.

### *OPRL* regulates the expression of *OPR* genes through the formation of RNA–DNA triplexes

The mechanisms of action of lncRNAs are diverse. They can act as signaling molecules to bind to transcription factors and participate in various regulatory processes or signaling pathways, thereby regulating the spatiotemporal expression of protein-coding genes [40]. Many lncRNAs reside in chromatin and can interact with proteins to promote or inhibit their binding to the target DNA region. Moreover, lncRNAs can serve as molecular scaffolds [8] and interact with various proteins to form ribonucleoprotein complexes. Specific sites within lncRNAs can bind to certain regulatory molecules, affecting several biological processes [41]. Some lncRNAs can act as molecular sponges for miRNAs, blocking their interaction with downstream target genes and indirectly regulating target gene function [42]. On the one hand, lncRNAs can be targeted by miRNAs to produce small interfering RNAs [42, 43]. On the other hand, lncRNAs serve as the source of miRNAs or regulate miRNA accumulation or activity at transcriptional and post-transcriptional levels [44]. In *trans*-lncRNAs, forming RNA–DNA triplets may represent a common feature of chromatin interaction for recognizing target genes by lncRNAs [45]. Using triad prediction software, we predicted that *OPRL*s and *OPR*s formed triple helices in the overlapping region of *OPRL*s located at the N-terminus (Supplementary Data Fig. S2). The band shift in the electrophoretic mobility shift assay indicated that *OPRLs* targeted chromatin by forming triple helices (Fig. 2C).

## Materials and methods

### Plant materials and chemical reagents

The *Camellia sinensis* var. *sinensis* cultivar ‘Zhongcha 108’ was used in this study and cultivated in the tea experimental field of Anhui Agricultural University (117.27 E; 31.86 N). Additionally, *N. benthamiana*, *A. thaliana* (Col-0), and *S. tuberosum* L. (cultivar ‘E Shu6’) plants were cultivated in a greenhouse under controlled conditions at 23°C with a 16/8-h photoperiod.

MeJA and DIECA, an inhibitor of JA synthesis, were purchased from Hefei Yili Biotechnology Co., Ltd. MeJA and DIECA were administered for exogenous hormone treatment at concentrations of 300 μM and 10 mM, respectively.

### Infection with pathogenic fungi

A pathogenic isolate (TYDY-2) belonging to *Colletotrichum camelliae* was used to infect the leaves of tea and potato plants. The TYDY-2 strain was initially cultured on potato glucose agar (PDA) medium at 28°C for 5 days, following which the spores were collected via centrifugation at 6000 xg/min for 10 min. Subsequently, the collected spores were resuspended in sterile water, and their concentration was adjusted to 106 spores/ml under microscopic monitoring for subsequent inoculation.

A total of 50 μl of the conidial suspension of TYDY-2 was then inoculated into plant leaves punctured at the upper epidermis using a sterile syringe. Control plants were inoculated with an equivalent volume of sterile distilled water. The inoculated leaves were covered with a film to maintain high humidity and facilitate fungal growth. Following a 24-h incubation period, the film was removed, and any symptoms on the infected leaves were recorded within 6 days post-inoculation. Subsequent to symptom observation, the infected leaves were collected and rapidly frozen. These frozen samples were then used to detect mRNA and lncRNA expression levels through RNA-sequencing (RNA-seq) and qRT–PCR.

### Detection of mRNAs and lncRNAs in tea plants using RNA-seq

Transcriptomic sequencing of mRNAs and lncRNAs in TYDY-2-infected leaves was conducted by the Beijing Genomics Institute (BGI). Total RNA was isolated from each sample, with ribosomal RNA removed prior to constructing a chain-specific library. All reconstructed transcripts were matched and aligned with reference genomic data (http://139.196.163.62/). The lncRNAs were identified using CPC software, txCdsPredict software, CNCI software, and the pfam database.

### Acquisition of data from lncRNAs of different plant species

The cDNA sequences of lncRNAs from several plant species were downloaded from CANTATAdb, including *Malus domestica*, *S. tuberosum*, *Theobroma cacao*, *Populus trichocarpa*, *Oryza nivara*, *Corchorus capitularis*, *Prunus persica*, *M. truncatula*, *Glycine max*, *Vitis vinifera*, and *A. thaliana*. Target gene sequences of lncRNAs were screened and downloaded from the National Center for Biotechnology Information (NCBI, https://www.ncbi.nlm.nih.gov). Links to various database sites are listed in Supplementary Data Table S4.

Data on the chromosomal location of *OPR*s and *OPRL*s across different species, including angiosperms and lower plants, was extracted from the abovementioned plant genome websites (NCBI, https://www.ncbi.nlm.nih.gov; CANTATAdb, http://cantata.amu.edu.pl/index.html).

### Gene cloning, qRT–PCR, multiple sequence alignment, and phylogenetic analysis

Total RNA was extracted from tea and potato plant leaves using Fruit-mate (Takara, Dalian, China) and the RNAiso Plus kit (Takara, Dalian, China), followed by reverse transcription to cDNA using PrimeScript-RT-Master Mix (Takara). PCR amplification was conducted using PrimeSTAR Max DNA polymerase (Takara). Real-time PCR was performed using Hieff qPCR SYBR Green Mix (Yeasen). The primer sequences used for PCR are listed in Supplementary Data Table S5.

The PCR products were ligated into the pEASY Blunt Simple Vector (TransGen Biotech, Beijing, China) and transformed into chemically competent DH5α cells (Weidi Biotechnology, Shanghai, China) for subsequent sequence analysis.

Multiple sequence alignment was conducted using DNAMAN software. A phylogenetic tree was constructed (using adjacency statistics) using MEGA 6.0 with a bootstrap test of 1000 replicates. The evolutionary distance was calculated using the Poisson model.

### Plasmid construction and heterologous expression of *CsOPR9-1* and *CsOPRL* in *A. thaliana*

*CsOPR9-1* and *CsOPRL* were expressed in *A. thaliana*, and hybrid plants were generated through crossing. Primer sequences with the attB linker were ligated to the complete open reading frames of *CsOPR9-1* and *CsOPRL* via *in vitro* PCR amplification. The resulting PCR products were purified using the Gateway BP cloning enzyme mixture, cloned into the entry vector pDONR207 (laboratory of Xiangchengbin, USTC), and then transferred into the expression vector pCB2004 (laboratory of Xiangchengbin, USTC) using the Gateway LR cloning enzyme system. The expression vectors containing the target genes (pCB2004-*CsOPR9-1* and pCB2004-*CsOPRL*) were subsequently introduced into the expression strain GV3101 (laboratory of Xiangchengbin, USTC). The primer sequences are listed in Supplementary Data Table S5.

The recombinant plasmids pCB2004-*CsOPR9-1* and pCB2004-*CsOPRL* were chemically transformed into GV3101. Plants overexpressing *CsOPR9-1* and *CsOPRL* were generated via *Agrobacterium*-mediated transformation of WT *A. thaliana* (Col-0). Following homozygous culture to the *T*_3_ generation, plants overexpressing *CsOPR9-1* and *CsOPRL* were hybridized to produce hybrid plants.

### Electrophoretic mobility shift assay

The electrophoretic mobility shift assay was conducted to detect the triplex formation of *CsOPR9-1* with *CsOPRL*, following a previously reported method with some modifications [46]. Single nucleotide strands of the predicted triple helix formation site in *CsOPR9-1*, the RNA strand with the specific sequence of the predicted binding domain in *CsOPRL*, and control RNA were procured from General Biosystems Company. Initial double-stranded hybridization involved single-stranded cDNA in hybridization buffers (10 mM Tris–HCl + 50 mM NaCl +10 mM MgCl_2_; pH 7.4) at 95°C for 5 min and then cooled to room temperature. Subsequently, 200 nM double-stranded DNA was incubated with single strands of RNA at varying concentrations in a hybridization buffer at 60°C for 1 h and then cooled to room temperature to form a triple helix. The 4-μl reaction mixture was analyzed on 15% polyacrylamide gels and stained with GelRed for 35 min. The triplex sequences are listed in Supplementary Data Table S5.

### Use of *Agrobacterium*-mediated transient transformation in *N. benthamiana* to verify the relationship between *OPRs* and *OPRLs*

As previously described, tobacco leaves underwent *Agrobacterium*-mediated co-transformation to confirm the regulatory relationship between lncRNAs and mRNAs [47]. Overnight-grown transformed *Agrobacterium* strains (pCB2004-*CsOPRL*, pCB2004-*StOPRL1*, *CsOPR9-1*-HA, and *StOPR10-4*-HA) were injected into the leaves of *N. benthamiana* when the OD at 600 nm reached 0.8. The cultures were centrifuged, and the resulting pellet was resuspended twice in a solution containing 10 mM MgCl_2_, 10 mM MES, and 100 μM acetosyringone (pH 5.6). The suspension cultures were then injected into the leaves of well-watered 6-week-old plants. Leaf samples were harvested 2 days post-injection, and gene expression was analyzed via western blotting and qRT–PCR, as described in the sections above. Each experiment was repeated at least three times.

### Antisense oligonucleotide-mediated gene suppression and infection of tea plants with pathogenic fungi

Candidate AsODNs targeting *CsOPRL* and *CsOPR9-1/2/3/4* were selected using SOLIGO software and synthesized by General Biosystems Company. A total of 1 ml of 100-μM AsODN solution was injected into tea seedlings, while tea seedlings injected with sense oligonucleotides (sODNs) served as control. Further experiments were conducted after 48 h of incubation, or the samples were frozen in liquid nitrogen for quantitative gene expression analysis. All primer sequences are listed in Supplementary Data Table S5.

Subsequently, tea plants were infected with pathogenic fungi following a 24-h incubation period, and symptoms were observed after an appropriate number of days.

### *Agrobacterium*-mediated transient gene expression and infection of tea plants with pathogenic fungi

*Agrobacterium*-mediated transient gene expression was induced in tea plants following the protocol described in a previous study [12]. Cultured *Agrobacterium* strains (pCB2004 *CsOPRL*, *CsOPR9-1*-HA) were grown overnight until the OD at 600 nm reached 0.8. The bacterial cells were injected into the leaves of tea plants and resuspended twice in the suspended solution, as described previously. After a 2-h incubation at room temperature, the tea leaves were injected with the bacterial cells and grown in a greenhouse for 72 h. Subsequently, quantitative analysis was performed to evaluate gene expression.

Following a 24-h incubation period, tea plants were infected with pathogenic fungi, and symptoms were observed after an appropriate number of days.

### Knockout of *StOPRL1* via CRISPR/Cas9 technology

A specific genomic RNA (gRNA) targeting *StOPRL1* was selected using the online tool CRISPR-P (http://cbi.hzau.edu.cn/crispr/). The target sgRNA expression box was constructed using the intermediate vector pYLgRNA-AtU3d and assembled into the pYLCRISPR/Cas9 vector. The recombinant plasmid, confirmed by correct sequencing results, was transformed into *Escherichia coli*. Subsequently, the recombinant plasmid was transformed into *Agrobacterium* for further experimentation.

The CRISPR/Cas9-*StOPRL1* expressing plasmid was transformed into potato via *Agrobacterium*-mediated transformation [48]. Transgenic potato plants were selected based on hygromycin resistance. DNA from positive plants was extracted for specific fragment amplification and subsequently sent for sequencing. The sequencing results were analyzed using SnapGene software. All primer sequences are listed in Table S5.

## Acknowledgements

We thank the National Natural Science Foundation of China (32372756, U21A20232).

## Authors contributions

T.J. performed the experiments. T.J. and T.M.J. analyzed the data. T.T.L. and C.L. provided experimental materials and tested methods. Y.B.H. performed data analysis. T.J. and T.M.J. contributed to the acquisition of reagents and materials and the selection of analytical tools. L.P.G. and T.X. guided the drawing of experimental figures. T.J. drafted the manuscript. Ting Jiang designed the experiments. All authors have read and approved the final manuscript.

## Data availability

The raw sequencing data from this study have been deposited in the Genome Sequence Archive in the BIG Data Center (https://bigd.big.ac.cn/), Beijing Institute of Genomics (BIG), Chinese Academy of Sciences, under the accession number CRA014289.

## Conflict of interest

The authors declare no competing interests.

## Supplementary data

Supplementary data are available at *Horticulture Research* online.

## Supplementary Material

Web_Material_uhae129
