# Supplementary figures and images for: Evolutionarily conserved 12-oxophytodienoate reductase trans-lncRNA pair affects disease resistance in tea (Camellia sinensis) via the jasmonic acid signaling pathway
Source: Hortic Res. 2024 May 6;11(7):uhae129. doi: 10.1093/hr/uhae129 (PMC11220176; doi:10.1093/hr/uhae129)

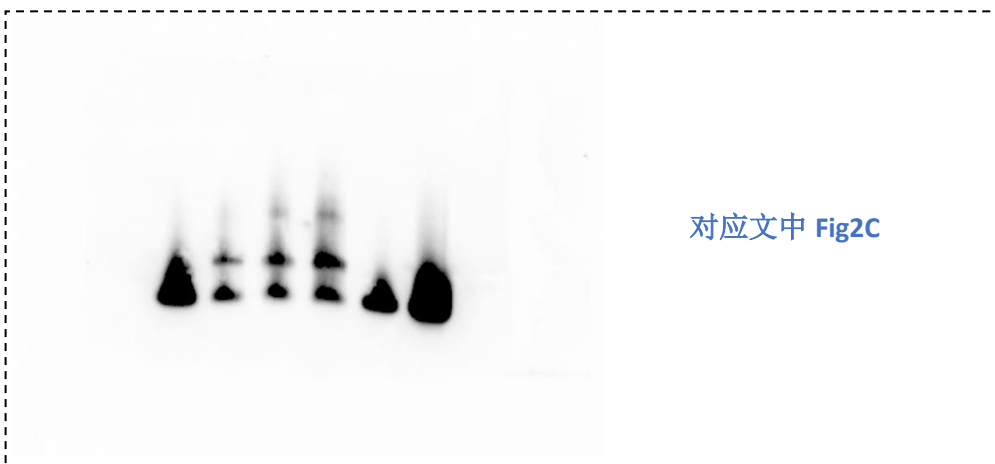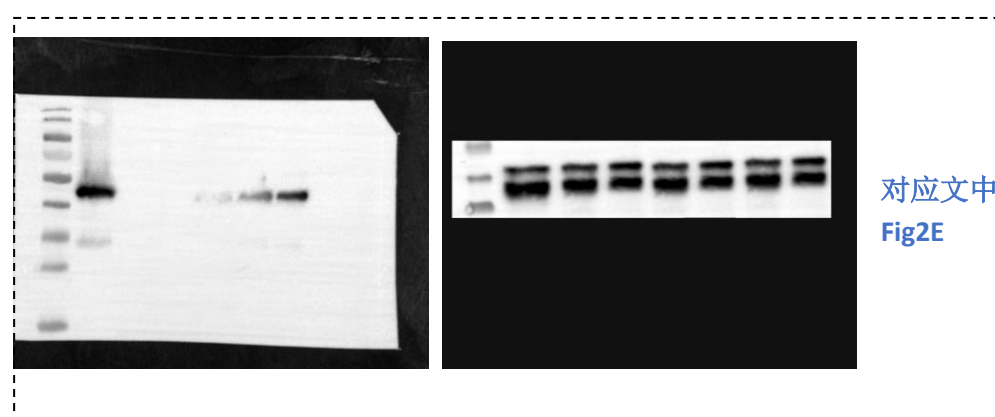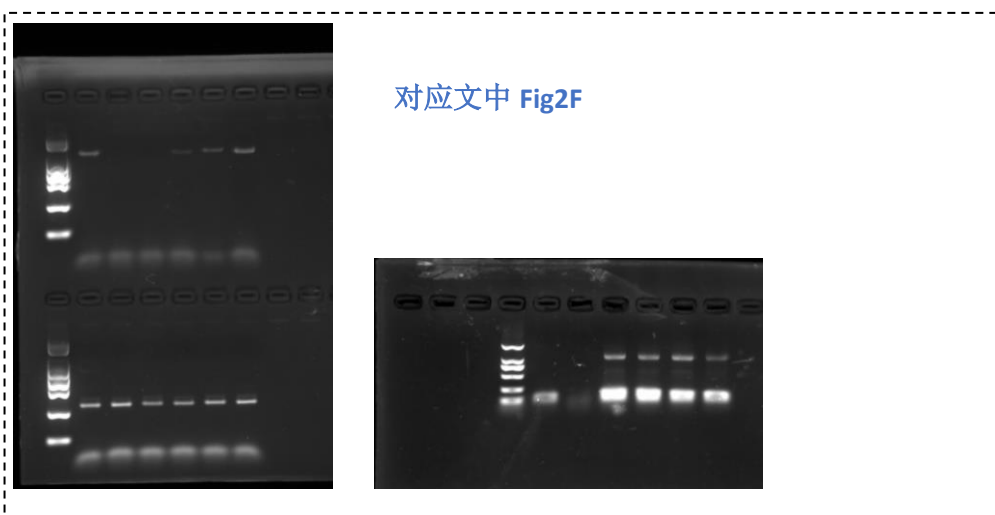

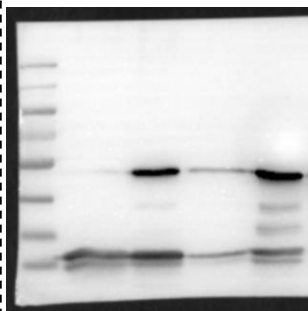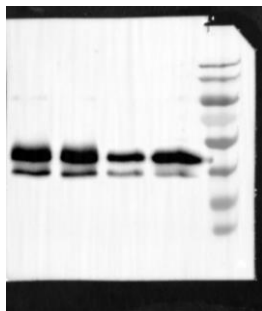

对 应 图 中  
Fig2H

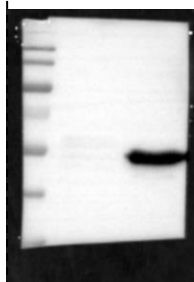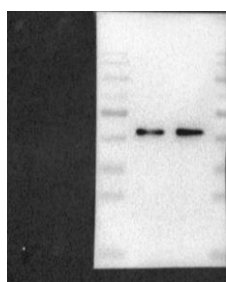

对应图中 Fig4C

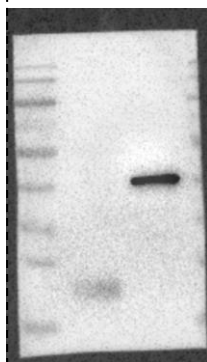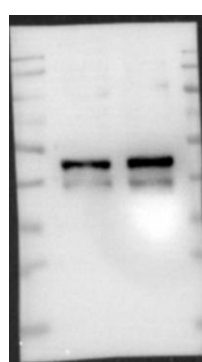

对应图中 Fig4D

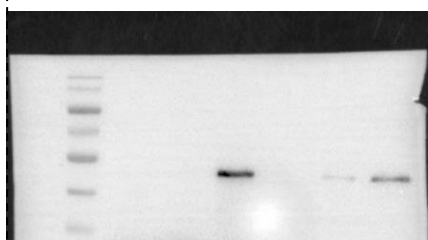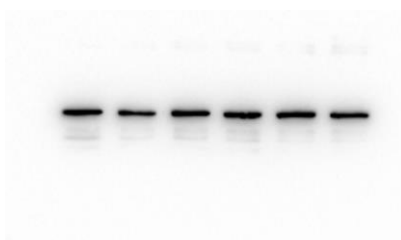

对应文中 Fig5A

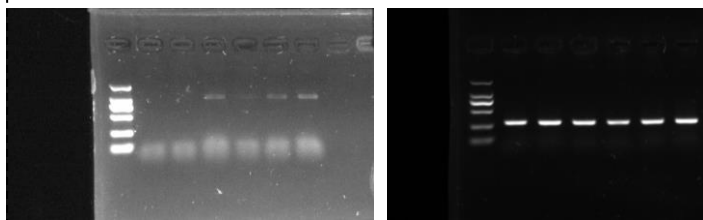

对应文中 Fig5B

Supplement: Web_Material_uhae129 [file web_material_uhae129.zip › western blot and RT PCR.pdf]
